# Dual EGFR blockade with cetuximab and erlotinib combined with anti-VEGF antibody bevacizumab in advanced solid tumors: a phase 1 dose escalation triplet combination trial

**DOI:** 10.1186/s40164-020-00159-1

**Published:** 2020-04-20

**Authors:** Vivek Subbiah, Ecaterina Ileana Dumbrava, Yunfang Jiang, Kyaw Z. Thein, Aung Naing, David S. Hong, Siqing Fu, Sarina A. Piha-Paul, Apostolia M. Tsimberidou, Filip Janku, Funda Meric-Bernstam, Razelle Kurzrock, Gerald Falchook

**Affiliations:** 1 Department of Investigational Cancer Therapeutics, The University of Texas MD Anderson Cancer Center, Houston, TX USA; 2 Center for Personalized Cancer Therapy and Division of Hematology and Oncology, University of California San Diego Moores Cancer Center, La Jolla, CA USA; 3 Sarah Cannon Research Institute at HealthONE, Denver, CO USA; 4 Department of Investigational Cancer Therapeutics (Phase I Clinical Trials Program), Division of Cancer Medicine, Unit #455, University of Texas MD Anderson Cancer Center, 1515 Holcombe Blvd, Houston, TX 77030 USA

**Keywords:** Dual EGFR blockade, Cetuximab, erlotinib and bevacizumab, Advanced solid tumors, Phase 1 dose escalation

## Abstract

**Background:**

Angiogenesis and activation of the epidermal growth factor (EGFR) pathway play an essential role in tumor proliferation and metastasis. Targeting angiogenesis or EGFR alone does not yield adequate tumor control in most solid tumors. Overcoming intrinsic and/or acquired resistance may need a doublet or triplet therapy strategy. Herein, we report the safety and feasibility of dual EGFR blockade with EGFR monoclonal antibody and EGFR tyrosine kinase inhibitor combined with anti-VEGF antibody in advanced solid tumors.

**Methods:**

We conducted a phase I study combining erlotinib, cetuximab, and bevacizumab. Patients with advanced or metastatic solid tumors (excluding colorectal and non-small cell lung cancers) were analyzed for safety, toxicity profile, and response. Anti-tumor activity was evaluated per response evaluation criteria in solid tumors (RECIST 1.0).

**Results:**

Thirty-six patients received treatment on a range of dose-levels. The most frequent tumor types enrolled were cervical (n = 10), head and neck squamous cell (n = 10), and follicular thyroid (n = 4) cancers. The most common treatment-related grade ≥ 2 adverse events were rash (56%), hypomagnesemia (17%), pruritus (11%), diarrhea (8%), and tumor-related bleeding (8%). Seventeen of 19 patients (89%) treated at the maximum tolerated dose did not present treatment-related dose-limiting toxicity. Fifteen (63%) of the 24 evaluable patients achieved a disease control (stable disease ≥ 4 months (n = 14) and partial response (n = 1). The median number of prior lines of therapies was 3 (range 1–10).

**Conclusions:**

The triplet combination of erlotinib, cetuximab, and bevacizumab was well tolerated, conferring clinical benefit in heavily pretreated patients. Future studies are warranted with second or third-generation EGFR tyrosine kinase triplet combinations in the EGFR pathway aberrant patients.

*Trial Registration:* ClinicalTrials.gov Identifier: NCT00543504. Sponsor(s): National Cancer Institute (NCI), MD Anderson Cancer Center

## Background

Genome driven precision oncology has primarily been focused on monotherapy for single-gene alterations [1]. While this has led to many successful targeted therapies [2–4], resistance to targeted therapies develop. One strategy to manage innate and acquired resistance is combination therapies with other targeted agents. Resistance to *BRAF*^*V600E*^ in BRAF monotherapy was overcome by combining BRAF and MEK inhibition in melanoma [5–7]. Similarly, combined inhibition was successful in patients with non-small cell lung cancer (NSCLC) and anaplastic thyroid cancer, that led to US Federal Drug Administration (FDA) approval in these diseases. Contemporaneously, EGFR was identified as an innate resistance mechanism in BRAF V600E positive colorectal cancer (CRC). A triplet combination of epidermal growth factor receptor (EGFR) monoclonal antibody and BRAF + MEK inhibitors showed clinical benefit [8]. In addition, recent precision oncology studies like WINTHER and I-PREDICT used customized combination strategies to address multiple pathways [9, 10]. The first iteration of the NCI-MATCH, National Cancer Institute-Molecular Analysis for Therapy Choice, or EAY131, a phase II precision medicine trial, sought to determine whether matching certain drugs in adults whose tumors have specific gene abnormalities will effectively treat their cancers, regardless of tumor types. The second-generation NCI-match planned is the combo-match for doublet therapies that tests combination therapy targeting.

Activation of the EGFR pathway plays a vital role in tumor proliferation of several solid tumors [11]. Cetuximab, a monoclonal antibody against EGFR, is commonly used in CRC [12, 13] and head and neck squamous cell cancers (HNSCC) [14, 15]. Erlotinib, a first-generation EGFR tyrosine kinase inhibitor is approved for the treatment of NSCLC [16, 17]. Preclinical studies showed that combination of monoclonal antibodies and tyrosine kinase inhibitors synergistically inhibit the growth of NSCLC and CRC cell lines [18–20].

Angiogenesis, mediated by the vascular endothelial growth factor receptor (VEGFR) and its ligands (VEGF), is critical for tumor growth and metastasis [21]. Bevacizumab is a recombinant anti-VEGF monoclonal antibody and is approved alone or in combination with chemotherapy for treatment of CRC, NSCLC, glioblastoma, cervical, ovarian, and renal cell cancers [22–26].

Furthermore, clinical and pre-clinical studies show that the combination of anti-VEGF and anti-EGFR therapy yields improved response rate and survival [27, 28]. The synergistic activity of the combination might be explained by the fact that acquired resistance to EGFR inhibitors is partially due to activation of the VEGF signaling pathway [29, 30]. Herein, we report the feasibility and safety results of a single-center triplet combination of anti-VEGF (bevacizumab) and dual EGFR inhibition (erlotinib, cetuximab) in patients with advanced or metastatic solid tumors.

## Methods

This is an investigator-initiated, single-center phase I clinical trial that employed a 3 + 3 dose-escalation design. The primary endpoints were to determine the maximum tolerated dose (MTD) and dose-limiting toxicities (DLT) of bevacizumab in combination with erlotinib and cetuximab. We also evaluated the anti-tumor efficacy of this treatment per response evaluation criteria in solid tumors (RECIST 1.0) [31].

The study was conducted at The University of Texas M. D. Anderson Cancer Center (MDACC) per Institutional Review Board guidelines. The results of the phase I study for tumor-specific cohorts were previously reported for CRC and NSCLC [32, 33]. The study accrual period was from October 2007 to August 2013. The patients reported herein included all patients with heavily pre-treated advanced solid tumors as part of a dose-escalation study conducted in patients with advanced cancer. The dose-escalation portion of the study determined the recommended phase II dose (RP2D) to be bevacizumab 10 mg/kg IV every 2 weeks; cetuximab loading 400 mg/m^2^, maintenance 250 mg/m^2^ IV weekly; and erlotinib 150 mg PO daily. The cycle was 28 days. Patients were treated at variable dose levels, depending on the time of study entry (Table 1).

**Table 1:** Patient characteristics

Characteristics	Number of patients n = 36, n (%)
Age (years)	
Median	54
Range	(15–79)
Gender	
Male	14 (39%)
Female	22 (61%)
Race	
White/Caucasian	28 (78%)
Black/African-America	4 (11%)
Other	4 (11%)
Smoking	
Active or history of smoking	18 (50%)
Never smoker	18 (50%)
ECOG performance status	
0	1 (3%)
1	30 (83%)
2	5 (14%)
Tumor type	
Head and neck squamous cell carcinoma	10 (28%)
Cervical cancer	10 (28%)
Thyroid follicular carcinoma	4 (11%)
Breast cancer	3 (8%)
Pancreatic cancer	3 (8%)
Salivary glands cancer	2 (6%)
Bladder urothelial carcinoma	2 (6%)
Sarcoma	1 (3%)
Vulvar cancer	1 (3%)
Squamous cell carcinoma of the skin	1 (3%)
Number of prior systemic therapies	
Median	3
Range	1–10
Prior systemic treatment with anti-EGFR	8 (22%)
Prior systemic treatment with anti-VEGF	9 (25%)
Prior systemic treatment with anti-EGFR and anti-VEGF (sequential)	2 (6%)

Patients had metastatic or advanced solid tumor not amendable to standard therapy, an Eastern Cooperative Oncology Group (ECOG) performance status 0–2, and adequate hematologic, hepatic, and renal function. Exclusion criteria included hemoptysis, unexplained bleeding, significant cardiovascular disease, intercurrent uncontrolled illness, significant gastrointestinal bleeding within 28 days, hemorrhagic brain metastases, prior abdominal surgery within 30 days, pregnancy, and a history of hypersensitivity to bevacizumab, cetuximab, and/or erlotinib. Treatment with prior cytotoxic therapies must have ended at least 3 weeks before enrollment, and biologic treatment must have completed at least 2 weeks or five drug half-lives before enrollment (whichever is shorter).

### Statistical analysis

No formal hypotheses were tested, and analyses were descriptive and exploratory. Non-parametric correlations were determined with Spearman’s rank correlation coefficient.

## Results

A total of 36 patients with advanced or metastatic solid tumors received treatment on a range of dose-levels. The most frequent tumor types enrolled were; cervical (n = 10), HNSCC (n = 10), and follicular thyroid (n = 4) cancers. The MTD and the RP2D was determined to be the FDA-approved doses for all three drugs (erlotinib 150 mg orally daily, cetuximab 400 mg/m^2^ loading dose, then 250 mg/m^2^ intravenous (IV) weekly and bevacizumab 10 mg/kg IV every 2 weeks). This combination was safe and well tolerated.

Out of the 19 patients treated at the RP2D, 7 patients (37%) required a dose reduction because of grade 2–3 skin rash (n = 6) and grade 3 elevated liver enzymes (n = 1). The most frequent treatment-related grade ≥ 2 adverse events likely related to the EGFR inhibition by cetuximab and erlotinib were: rash (56%), hypomagnesemia (17%), pruritus (11%), diarrhea (8%) and likely related to antiangiogenic effect of bevacizumab were: hypertension, bleeding, and fistula (Table 2).

**Table 2:** Treatment-related grade ≥ 2 adverse events

	Dose level	1 n = 1	2 n = 2	3 n = 0	4 n = 1	5 n = 6	6 n = 4	7 n = 3	8 n = 19	Total N = 36
	Bevacizumab IV q2w (mg/kg)	2.5	5	5	5	7.5	7.5	7.5	10	
	Cetuximab IV weekly (mg/m^2^)*	100, 75	100, 75	200, 125	200, 125	200, 125	400, 250	400, 250	400, 250	
	Erlotinib PO daily (mg)	50	50	50	100	100	100	150	150	
Rash										
Grade 2		0	0	0	1	3	2	0	10	16 (44%)
Grade 3		0	0	0	0	0	0	0	4	4 (11%)
Pruritus										
Grade 2		0	0	0	1	0	0	0	1	2 (6%)
Grade 3		0	0	0	0	0	0	0	1	2 (6%)
Diarrhea										
Grade 2		0	0	0	0	0	0	0	3	3 (8%)
Fatigue										
Grade 2		0	0	0	0	1	0	1	0	2 (6%)
Hand-foot syndrome										
Grade 2		0	0	0	0	0	0	1	0	2 (6%)
Hypomagnesemia										
Grade 2		0	0	0	0	0	0	0	2	2 (6%)
Grade 3–4		0	0	0	0	1	0	0	3	4 (11%)
Nausea/vomiting										
Grade 2		0	0	0	0	0	0	0	1	1 (3%)
Mucositis										
Grade 2		0	0	0	0	0	0	0	1	1 (3%)
Anorexia										
Grade 2		0	0	0	0	0	0	0	2	2 (6%)
Hypertension										
Grade 2		0	0	0	0	0	0	0	1	1 (3%)
Bleeding										
Grade 3		0	0	0	0	1	0	0	2	3 (8%)
Elevated AST/ALT										
Grade 3		0	0	0	0	0	0	0	1	1 (3%)
Anemia										
Grade 3		0	0	0	0	1	0	0	0	1 (3%)
Transvaginal fistula										
Grade 4		0	0	0	0	2	0	0	0	2 (6%)

* Cetuximab dose shown as loading dose and maintenance dose

**Fig. 1:**
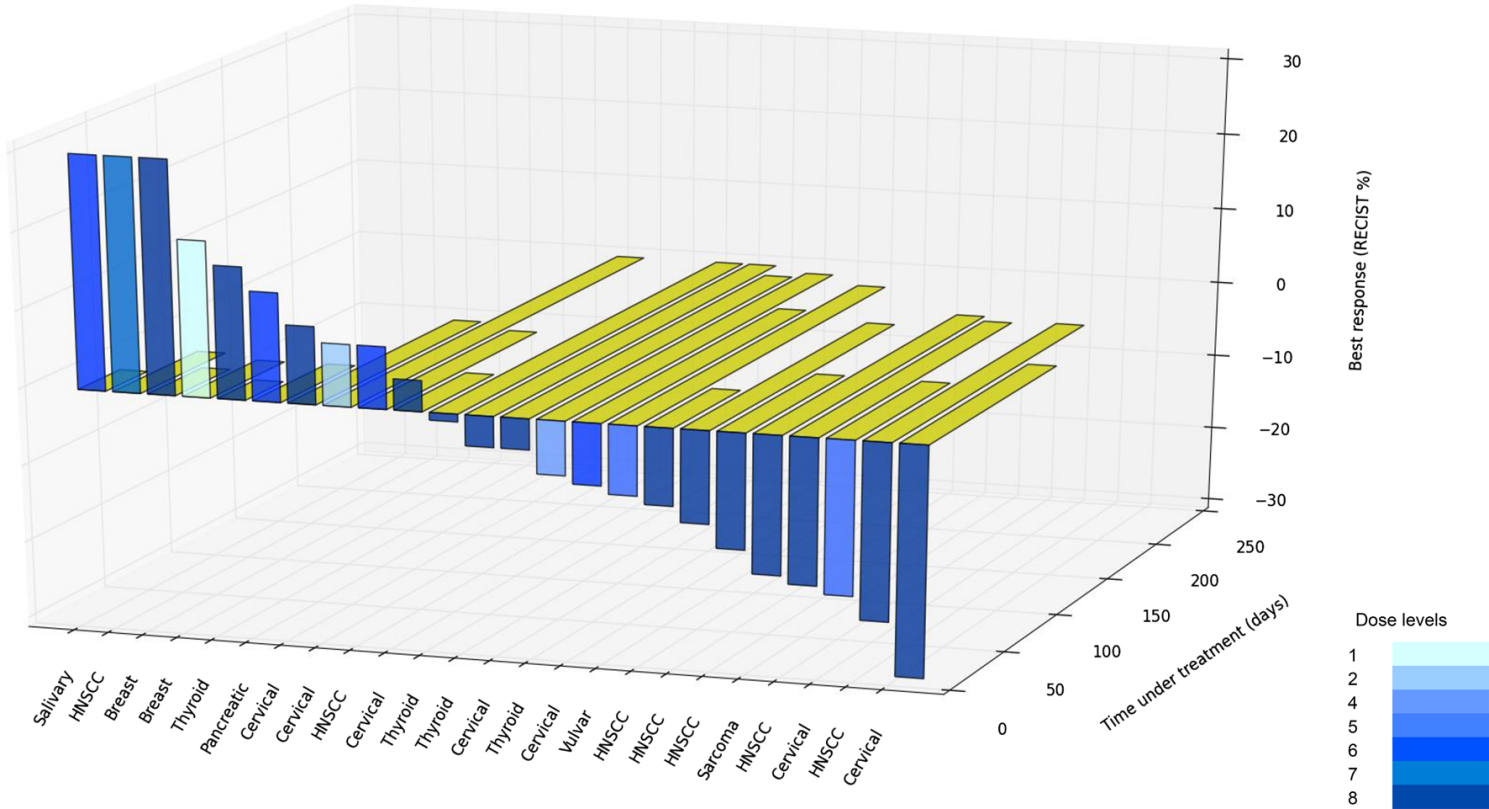
Best response and time under treatment (3D waterfall plot)

Of the 24 evaluable patients, 14 patients (58%) presented a disease control (defined as stable disease or partial response per RECIST 1.0 of more than 16 weeks), including patients who previously received bevacizumab, erlotinib and/or cetuximab (Fig. 1).

Although only one patient achieved a partial response, 14 patients had a clinical benefit and some durable disease control from the treatment. This might be related to the different pattern of the response of targeted therapies and antiangiogenics, and the radiologic criteria used (RECIST 1.0) has many limits in assessing the response to these treatments [34].

Exploratory analysis of mutations in EGFR, BRAF, KRAS, NRAS, MET, PIK3CA, and TP53 genes was done in a Clinical Laboratory Improvements Amendments (CLIA)-approved laboratory at MD Anderson Cancer Center on archived tissue.

Only two patients were identified to have EGFR mutations. One patient with epithelioid sarcoma had a pathogenic activating mutation in exon 18 (*EGFR p.G719D*) and achieved a stable disease per RECIST 1.0 for more than 6 months, with 18% decreased of the target lesions as compared with baseline. Another patient with salivary gland carcinoma with an *EGFR p.D770N* mutation (exon 20) showed no response to treatment and presented new metastases at the first restaging.

## Discussion

Dual EGFR blockade with EGFR monoclonal antibody and oral EGFR tyrosine kinase inhibitor was shown be additive or synergistic with predictable safety profile [35–37]. Cetuximab and erlotinib contributed to significant decrease in cellular proliferation without achieving substantial cell death, and enhanced shifting of cancer stem cells from mesenchymal states to epithelial phenotype, thereby reducing local invasion and metastasis in HNSCC cell lines [37]. Wheler and colleagues demonstrated that cetuximab and erlotinib combination was well tolerated and five out of 20 patients (25%) had achieved partial response and stable disease ≥ 6 months in patients with NSCLC [35].

VEGF and EGFR signaling pathways are intercorrelated; via up-regulating VEGF by EGFR expression and VEGF up-regulation independently contributing to EGFR resistance [29, 38–41]. Preclinical evidence suggested that inhibiting both pathways suppress AKT and ERK signaling and have notably shrunken the tumor growth in CRC cells lines [39]. In preclinical models and early phase trials, combination of VEGF and EGFR inhibition has shown activity in advanced solid tumors, including CRC, NSCLC, breast cancer, renal cell carcinoma and HNSCC [28, 42–44].

Recently, the results of a randomized, double-blind phase III study of erlotinib with ramucirumab (anti-VEGF therapy) or placebo in previously untreated EGFR-mutant metastatic non-small-cell lung cancer (RELAY) were reported, and the doublet therapy showed clinical benefit and results were positive [45]. Improving upon a doublet may warrant a triplet, and our trial shows safety, and feasibility of a triplet combination.

Falchook et al., had previously demonstrated the result of phase 1, dose-escalation study combining dual EGFR inhibition with anti-VEGF treatment in heavily pretreated patients with CRC [33]. Thirty-four percent had achieved either stable disease or partial response and most patients tolerated the regimen without dose-limiting toxicities. Hence, we are reporting the regimen in non-CRC and non-NSCLC cohorts (Fig. 1).

*EGFR* exon 20 insertions confer intrinsic resistance or lack of response to first-generation EGFR inhibitors such as erlotinib, compared to patients harboring other EGFR mutations [46, 47]. Also in preclinical models, exon 20 deletions have also been shown to confer resistance to cetuximab, while retaining sensitivity to other drugs such as poziotinib [48] and pan-ERBB inhibitors, such as neratinib and dacomitinib [49]. Robichaux and colleagues showed that first 11 patients with NSCLC carrying EGFR exon 20 mutations had achieved an objective response rate of 64% in a phase II trial [50]. Osimertinib and other third-generation EGFR inhibitors are still under investigation in patients with NSCLC harboring these mutations.

Although *PIK3CA* mutations in exon 20 (*H1047R*) have been identified as potential predictive biomarkers for non-response to cetuximab in KRAS-wild-type tumors, *PIK3CA* mutations in exon 9 have not been associated with resistance to EGFR inhibitors [51]. Interestingly all three patients who were found to have mutations in exon 9 (*E542K and E545K*) had a stable disease for more than 16 weeks.

There are several limitations of this study, including a small number of patients who had molecular testing, precluding from robust analysis. Since this was employed in advanced solid malignancies, EGFR mutation was not a criterion to enroll in the trial. However, our results show that combination of dual EGFR inhibition by erlotinib and cetuximab with bevacizumab is well-tolerated with the most common adverse event being manageable rash, in heavily pretreated patients with multiple solid tumors with a median of 3 prior systemic treatments. In addition, this trial was carried out in an era when comprehensive genomic panel was not routine in all patients. Moreover, results show the necessity of developing predictive biomarkers of treatment and integrating correlative studies in the clinical trials. Furthermore, not only the gene mutated is important, but also the annotation of each mutation within a gene. Functional annotation has become crucial in genomic medicine, and several algorithms have been developed.

With the advances of tumor DNA sequencing, there is a growing interest in personalized cancer therapy with genomically matched treatments and it would be suitable to explore the combination of a third-generation tyrosine kinase inhibitor targeting EGFR with cetuximab and bevacizumab in preselected patients with EGFR activating mutations and excluding patients with concomitant alterations that might confer resistance to the combination, such as KRAS mutations.

## Conclusions

Dual EGFR inhibition (erlotinib and cetuximab) combined with bevacizumab is a safe and well tolerated combination, demonstrating antitumor activity in patients with solid tumors, beyond CRC and NSCLC. Future studies are warranted with second or third-generation EGFR tyrosine kinase triplet combinations in the EGFR pathway aberrant patients. There is a critical need to develop and validate predictive biomarkers for genomically matched therapies and personalize cancer treatment.

## Lessons learned

1. Dual EGFR inhibition (erlotinib and cetuximab) combined with bevacizumab is a safe and well tolerated combination, demonstrating antitumor activity in patients with solid tumors beyond colorectal and non-small cell lung cancers
2. There is a critical need to develop and validate predictive biomarkers for genomically matched therapies and personalize cancer treatment

## Supplementary information

**Additional file 1.** Complete Statement of Competing Interest

## Data Availability

The datasets used and/or analyzed during the current study are available from the corresponding author on reasonable request.

## Abbreviations

EGFR: Epidermal growth factor
RECIST: Response evaluation criteria in solid tumors
FDA: US Federal Drug Administration
NCI-MATCH: National Cancer Institute-Molecular Analysis for Therapy Choice
CRC: Colorectal cancers
NSCLC: Non-small cell lung cancer
VEGF: Vascular endothelial growth factor
MTD: Maximum tolerated dose
DLT: Dose-limiting toxicities
CLIA: Clinical Laboratory Improvements Amendments
RP2D: Recommended phase II dose
ECOG: Eastern cooperative oncology group
