# Supplementary material for: Dual EGFR blockade with cetuximab and erlotinib combined with anti-VEGF antibody bevacizumab in advanced solid tumors: a phase 1 dose escalation triplet combination trial
Source: Exp Hematol Oncol. 2020 Apr 20;9:7. doi: 10.1186/s40164-020-00159-1 (PMC7171918; doi:10.1186/s40164-020-00159-1)
Supplement: Supplementary file 1 — Additional file 1. Complete Statement of Competing Interest [file 40164_2020_159_MOESM1_ESM.docx]

**Competing interests:**

**Funda Meric-Bernstam, MD**

**Consulting**

Aduro BioTech Inc., DebioPharm, eFFECTOR Therapeutics, F. Hoffman-La Roche Ltd., Genentech Inc., IBM Watson, Jackson Laboratory, Kolon Life Science, OrigiMed, PACT Pharma, Parexel International, Pfizer Inc., Samsung Bioepis, Seattle Genetics Inc., Tyra Biosciences, Xencor, Zymeworks

**Advisory Committee**

Immunomedics, Inflection Biosciences, Mersana Therapeutics, Puma Biotechnology Inc., Seattle Genetics, Silverback Therapeutics, Spectrum Pharmaceuticals

**Sponsored Research**

Aileron Therapeutics, Inc. AstraZeneca, Bayer Healthcare Pharmaceutical, Calithera Biosciences Inc., Curis Inc., CytomX Therapeutics Inc., Daiichi Sankyo Co. Ltd., Debiopharm International, eFFECTOR Therapeutics, Genentech Inc., Guardant Health Inc., Millennium Pharmaceuticals Inc., Novartis, Puma Biotechnology Inc., Taiho Pharmaceutical Co.,

**Honoraria**

Chugai Biopharmaceuticals, Mayo Clinic, Rutgers Cancer Institute of New Jersey, UT Health San Antonio,

**Dr. Aung Naing**

Research funding from NCI; EMD Serono; MedImmune; HealiosOnc. Nutrition; Atterocor; Amplimmune; ARMO BioSciences; Eli Lilly; KaryopharmTherapeutics; Incyte; Novartis; Regeneron; Merck; BMS; Pfizer, CytomXTherapeutics; Neon Therapeutics; CalitheraBiosciences; TopAllianceBiosciences; Kymab; PsiOxus; Immune Deficiency Foundation (Spouse)

On advisory board of CytomXTherapeutics, Novartis, Kymab, Genome

Travel and accommodation expense from ARMO BioSciences

**COI’s**

**Clinical Trial Research Support (paid to the institution)**

AstraZeneca; Abbisko; Anaeropharma Science; Arrien Pharmaceuticals; BeiGene; BioAtla, LLC; Boehringer Ingelheim; Eli Lilly & Co.; Hookipa Biotech GmBH; Huya Bioscience International; IMV, Inc.; Innovent Biologics, Co., Ltd.; Lyvgen Biopharm, Co., Ltd.; MacroGenics; Medivir AB; Millennium Pharmaceuticals, Inc.; Nerviano Medical Sciences; NeuPharma, Inc.; NIH/NCI; Novartis; OncoMed Pharmaceuticals; Parexel International, LLC; Sellas Life Sciences Group; Soricimed Biopharma, Inc. ; Tolero Pharmaceuticals

**Dr. Filip Janku**

- **Grant/Research Funding (Institutional)**: Novartis, Genentech, BioMed Valley Discoveries, Plexxikon, Deciphera, Piqur, Symphogen, Bayer, FujiFilm Corporation and Upsher-Smith Laboratories, Astex, Asana, Astellas, Agios, Proximagen, Bristol-Myers Squibb (all >$25,000)
- **Scientific Advisory Board**: Deciphera, IFM Therapeutics, Synlogic, Guardant Health, Ideaya, PureTech Health (<$25,000)
- **Paid Consultant**: Trovagene, Immunomet (>$25,000), Jazz Pharmaceuticals, Sotio (<$25,000)
- **Ownership Interests**: Trovagene (<$25,000)

**Dr. Razelle Kurzrock** has the following disclosure information: S

tock and Other Equity Interests

(IDbyDNA, CureMatch, Inc., and Soluventis); Consulting or Advisory Role (Gaido, LOXO,

X-Biotech, Actuate Therapeutics, Roche, NeoMed, Soluventis, Pfizer, and Merck); Speaker’s fee (Roche); Research Funding (Incyte, Genentech, Merck Serono, Pfizer, Sequenom, Foundation Medicine, Guardant Health, Grifols, Konica Minolta, DeBiopharm, Boerhringer Ingelheim, and OmniSeq [All institutional]); Board Member (CureMatch, Inc., and CureMetrix, Inc.).

**Gerald Steven Falchook**

Royalties (self): Wolters Kluwer (2014-present)

Advisory role (to institution): Fujifilm (2018)

Advisory role (self): EMD Serono (2010, 2011)

Travel (self): Bristol-Myers Squibb, EMD Serono (2011, 2012, 2013), Fujifilm (2018), Millennium (2013), Sarah Cannon Research Institute

Speakers bureau: Total Health Conferencing (2019)

Research funding [to institution, for any trial for which I have been the PI (ever) or subinvestigator (minimum last 4 years)]:

3-V Biosciences, Abbisko, Abbvie, ADC Therapeutics, Aileron, American Society of Clinical Oncology, Amgen, ARMO, AstraZeneca, BeiGene, Bioatla, Biothera, Celldex, Celgene, Ciclomed, Curegenix, Curis, Cyteir, Daiichi, DelMar, eFFECTOR, Eli Lilly, EMD Serono, Epizyme, Exelixis, Fujifilm, Genmab, GlaxoSmithKline, Hutchison MediPharma, Ignyta, Incyte, Jacobio, Jounce, Kolltan, Loxo, MedImmune, Millennium, Merck, miRNA Therapeutics, National Institutes of Health, Novartis, OncoMed, Oncothyreon, Precision Oncology, Prelude, Regeneron, Rgenix, Ribon, Strategia, Syndax, Taiho, Takeda, Tarveda, Tesaro, Tocagen, Turning Point Therapeutics, U.T. MD Anderson Cancer Center, Vegenics, Xencor

Dr. Hong's COI is below:

**David S Hong**

Research/Grant Funding:  AbbVie, Adaptimmune, Amgen, Astra-Zeneca, Bayer, BMS, Daiichi-Sankyo, Eisai, Fate Therapeutics, Genentech, Genmab, Ignyta, Infinity, Kite, Kyowa, Lilly, LOXO, Merck, MedImmune, Mirati, MiRNA, Molecular Templates, Mologen, NCI-CTEP, Novartis, Pfizer, Seattle Genetics, Takeda

Travel, Accommodations, Expenses: LOXO, MiRNA, ASCO, AACR, SITC, Genmab

Consulting or Advisory Role: Alpha Insights, Axiom, Adaptimmune, Baxter, Bayer, Genentech, GLG, Group H, Guidepoint Global, Infinity, Janssen, Merrimack, Medscape, Numab, Pfizer, Seattle Genetics, Takeda, Trieza Therapeutics

Other ownership interests: Molecular Match (Advisor), OncoResponse (founder), Presagia Inc (Advisor)

Vivek Subbiah:

**Research funding/ Grant support for clinical trials:**

Roche/ Genentech, Novartis, Bayer, GlaxoSmithKline, Nanocarrier, Vegenics, Celgene, Northwest Biotherapeutics, Berghealth, Incyte, Fujifilm, Pharmamar, D3, Pfizer, Multivir, Amgen, Abbvie, Alfa-sigma, Agensys, Boston Biomedical, Idera Pharma, Inhibrx, Exelixis, Blueprint medicines, Loxo oncology, Medimmune**,**  Altum, Dragonfly therapeutics, Takeda and, National Comprehensive Cancer Network, NCI-CTEP and UT MD Anderson Cancer Center, Turning point therapeutics, Boston Pharmaceuticals

**Travel:** Novartis, Pharmamar, ASCO, ESMO, Helsinn, Incyte,

**Consultancy/** **Advisory board:** Helsinn, LOXO Oncology/ Eli Lilly, R-Pharma US, INCYTE, QED pharma, Medimmune, Novartis.

Other: Medscape
